# Supplementary figures and images for: Factors associated with implant survival following total hip replacement surgery: A registry study of data from the National Joint Registry of England, Wales, Northern Ireland and the Isle of Man
Source: PLoS Med. 2020 Aug 31;17(8):e1003291. doi: 10.1371/journal.pmed.1003291 (PMC7458308; doi:10.1371/journal.pmed.1003291)

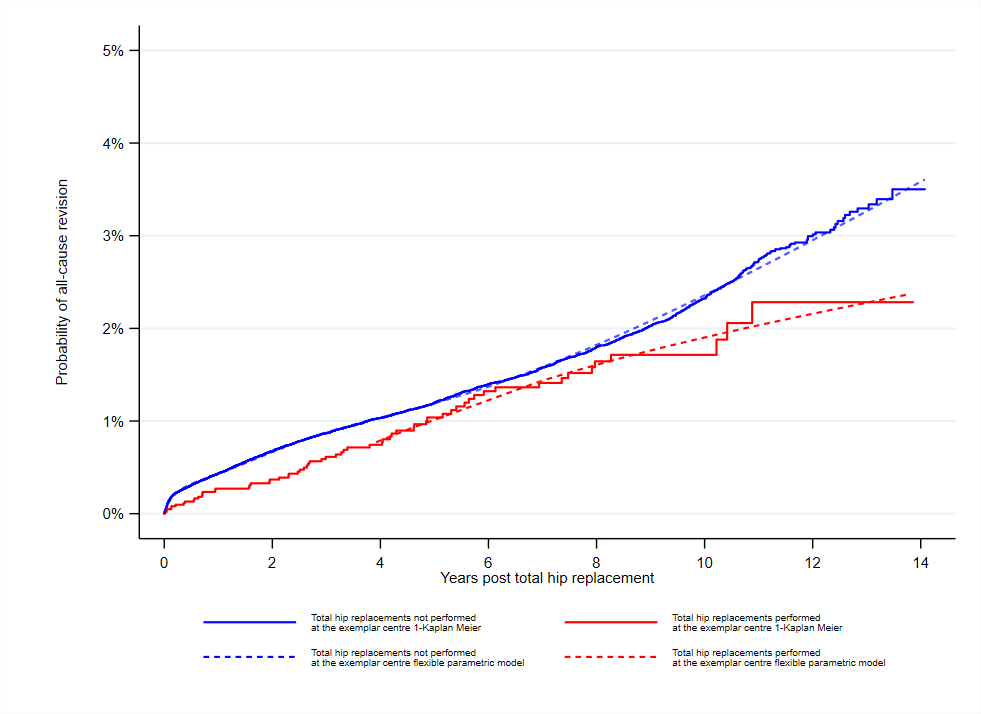

Supplement: S1 Fig — Goodness of fit was assessed visually using the above figure as well as by assessment of log-likelihood of different models. A model with 5 knots was chosen as further knots provided more complexity with little improvement in log-likelihood. (TIF) [file pmed.1003291.s002.tif]

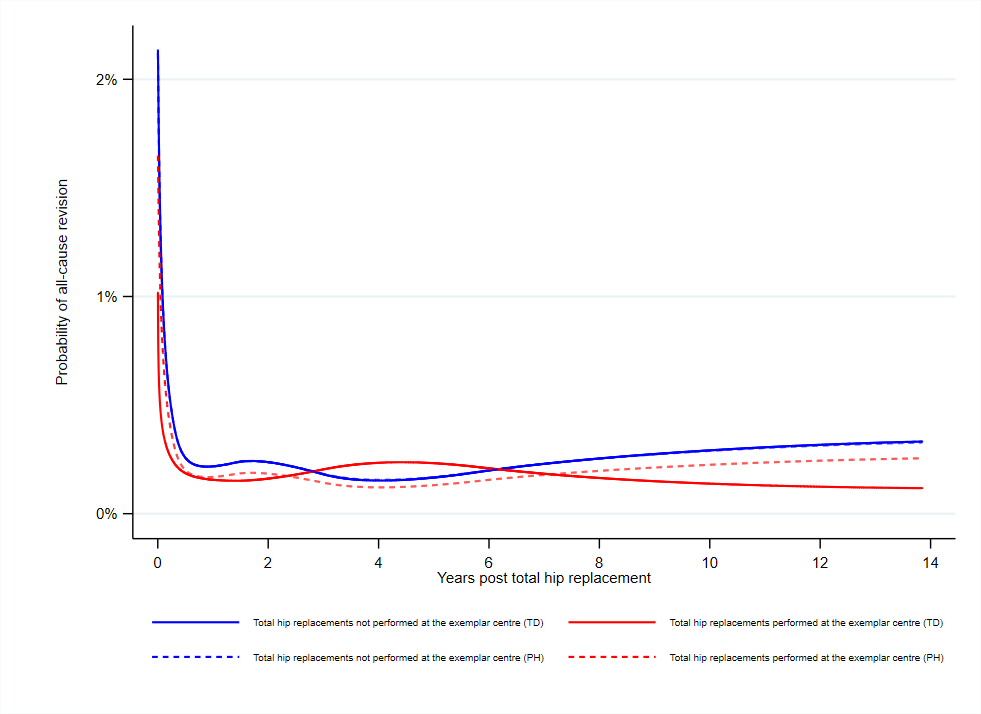

Supplement: S2 Fig — The above figure demonstrates that when using a flexible parametric survival analysis model that allows the hazard of failure to vary with time to compare TD and PH models. There is an apparent difference between the hazard of failure at the exemplar center and in all other units (the solid lines). The fact that these solid lines cross is highly indicative of the fact that the hazards are not proportional through the entire follow-up of the study. PH, proportional hazard; TD, time dependent. (TIF) [file pmed.1003291.s003.tif]

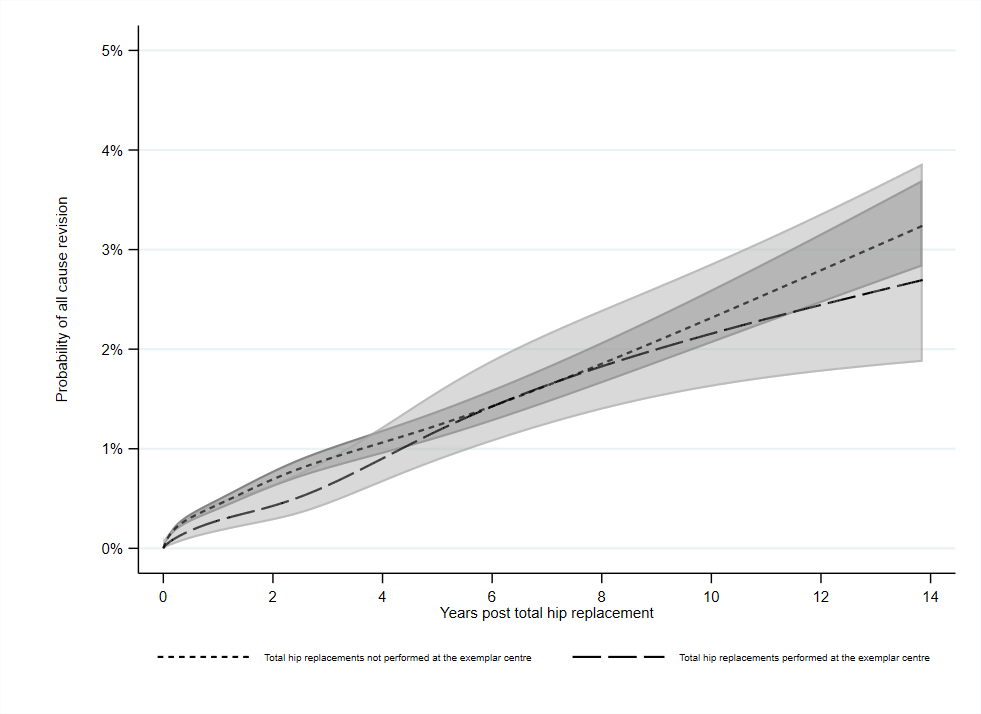

Supplement: S3 Fig — Results presented for a 68-year-old female patient with an American Society of Anesthesiology score of 2 and in the 10th decile of IMD organized by LSOA. FPSA, flexible parametric survival analysis; IMD, Index of Multiple Deprivation; LSOA, Lower Layer Super Output Area. (TIF) [file pmed.1003291.s004.tif]

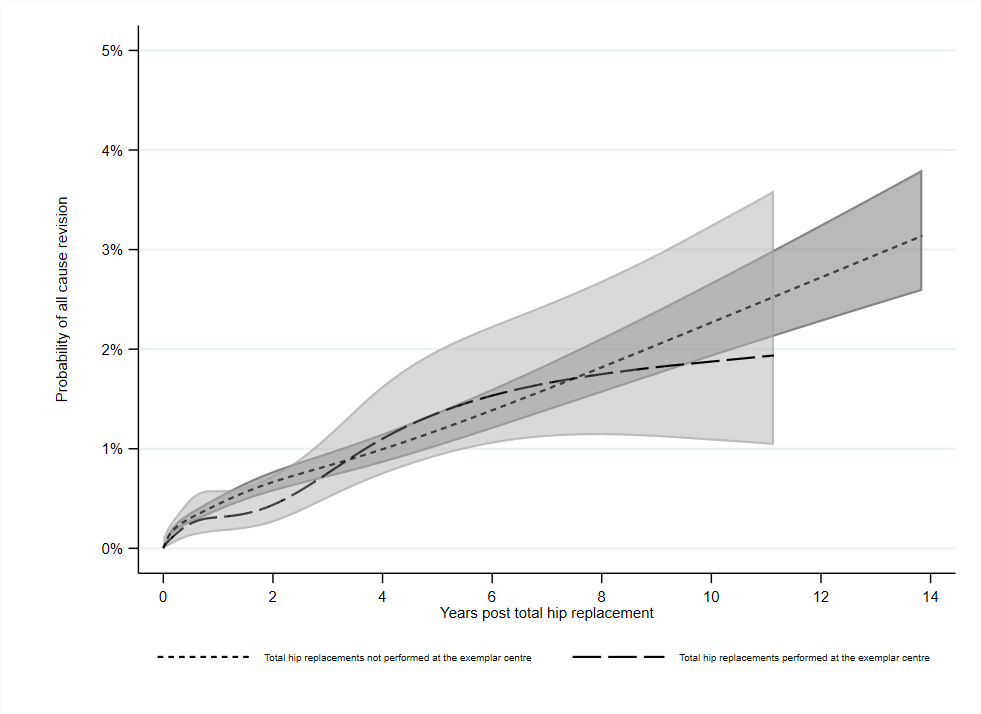

Supplement: S4 Fig — Results presented for a 68-year-old female patient with an American Society of Anesthesiology score of 2 and in the 10th decile of IMD organized by LSOA and body mass index in World Health Organization category 2. FPSA, flexible parametric survival analysis; IMD, Index of Multiple Deprivation; LSOA, Lower Layer Super Output Area. (TIF) [file pmed.1003291.s005.tif]

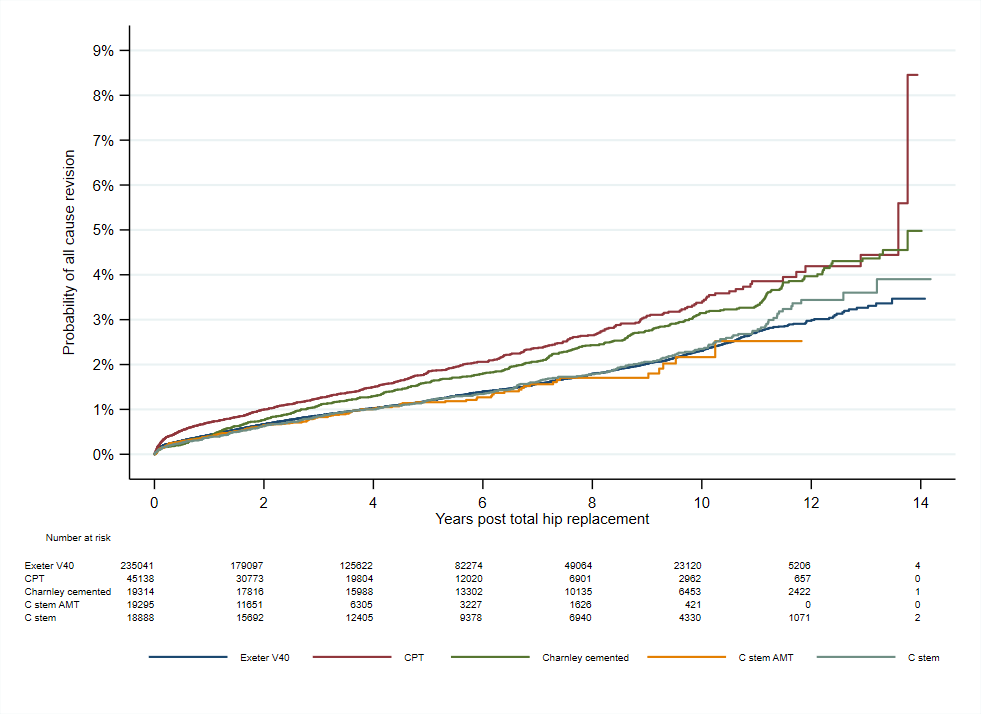

Supplement: S5 Fig — A comparison of the probability of all-cause revision (1 –Kaplan–Meier) for all constructs using the 5 most frequently implanted cemented femoral stems, demonstrating the differences in revision estimates between these stems. This suggests that the results demonstrated in this study may be achievable with other femoral stems. (TIF) [file pmed.1003291.s006.tif]
