# Supplementary material for: Factors associated with implant survival following total hip replacement surgery: A registry study of data from the National Joint Registry of England, Wales, Northern Ireland and the Isle of Man
Source: PLoS Med. 2020 Aug 31;17(8):e1003291. doi: 10.1371/journal.pmed.1003291 (PMC7458308; doi:10.1371/journal.pmed.1003291)
Supplement: S1 Table — A comparison of the distribution of BMI between the 2 exposure categories (Royal Devon & Exeter hospital and all other hospitals combined). BMI, body mass index. (DOCX) [file pmed.1003291.s007.docx]

|  | **Body Mass Index (kg/m^2^)** | **Overall** | **Royal Devon & Exeter** | **All other units** |
| --- | --- | --- | --- | --- |
| Underweight n (%) | <18.5 | 840 (0.5) | 34 (0.6) | 806 (0.5) |
| Normal Weight n (%) | 18.5 – 24.9 | 23,506 (15.2) | 874 (14.0) | 22,632 (15.3) |
| Overweight n (%) | 25 – 29.9 | 42,755 (27.7) | 1,581 (25.4) | 41,174 (27.8) |
| Class I Obesity n (%) | 30 – 34.9 | 27,013 (17.5) | 985 (15.8) | 26,028 (17.6) |
| Class II Obesity n (%) | 35 -39.9 | 9,480 (6.1) | 350 (5.6) | 9,130 (6.2) |
| Class III Obesity n (%) | >40 | 3,198 (2.1) | 126 (2.0) | 3,072 (2.1) |
| Missing Body Mass Index data n (%) | | 47,730 (30.9) | 2,277 (36.6) | 45,453 (30.7) |
| **Total** |  | **154,522** | **6,227** | **148,295** |
