# Supplementary material for: Factors associated with implant survival following total hip replacement surgery: A registry study of data from the National Joint Registry of England, Wales, Northern Ireland and the Isle of Man
Source: PLoS Med. 2020 Aug 31;17(8):e1003291. doi: 10.1371/journal.pmed.1003291 (PMC7458308; doi:10.1371/journal.pmed.1003291)
Supplement: S2 Table — Table detailing the distribution of missing data between the exposure categories (Royal Devon & Exeter hospital and all other hospitals combined). (DOCX) [file pmed.1003291.s008.docx]

|  | Exemplar centre n (%) | All other units n (%) | All other units restricted to same implants n (%) |
| --- | --- | --- | --- |
| Total | 6,227 | 658,531 | 148,295 |
| Missing age | 0 (0) | 0 (0) | 0 (0) |
| Missing sex | 0 (0) | 0 (0) | 0 (0) |
| Missing American Society of Anaesthesiology score | 0 (0) | 0 (0) | 0 (0) |
| Missing Socio-economic Status | 16 (0.3) | 39,223 (6.0) | 8,569 (5.8) |
| Missing Body Mass Index | 2,277 (36.5) | 247,742 (37.6) | 45,453 (30.7) |
