# Supplementary material for: Factors associated with implant survival following total hip replacement surgery: A registry study of data from the National Joint Registry of England, Wales, Northern Ireland and the Isle of Man
Source: PLoS Med. 2020 Aug 31;17(8):e1003291. doi: 10.1371/journal.pmed.1003291 (PMC7458308; doi:10.1371/journal.pmed.1003291)
Supplement: S3 Table — Table demonstrating the number of total hip replacements at risk at each time point following operation. For use in the interpretation of previous survival graphs. (DOCX) [file pmed.1003291.s009.docx]

|  | **Number of total hip replacements at risk (years following total hip replacement)** | | | | |
| --- | --- | --- | --- | --- | --- |
|  | **3** | **5** | **7** | **10** | **13** |
| All total hip replacements implanted in all other units | 422,263 | 285,468 | 172,630 | 61,395 | 8111 |
| All total hip replacements implanted in exemplar centre | 4,123 | 2,622 | 2,031 | 655 | 69 |
| Total hip replacement constructs using a cemented stem implanted in all other units | 245,755 | 171,881 | 112,251 | 44,610 | 6412 |
| Total hip replacement constructs using a cemented stem operated on at exemplar centre | 4,122 | 2,622 | 2,031 | 655 | 69 |
| Total hip replacement constructs using an Exeter V40 stem implanted in all other units | 147,596 | 10,051 | 62,715 | 22,465 | 2336 |
| Total hip replacement constructs using an Exeter V40 stem implanted in exemplar centre | 4,121 | 2,621 | 2,030 | 655 | 69 |
| Total hip replacement constructs using constructs used at exemplar centre implanted in all other units | 86,842 | 54,445 | 32,307 | 10,171 | 689 |
| Total hip replacement constructs using constructs used at exemplar centre implanted in exemplar centre | 4,121 | 2,621 | 2,030 | 655 | 69 |
| Total hip replacement constructs using an Exeter V40 stem implanted in all other units censoring revisions for instability and infection | 147,596 | 100,051 | 62,715 | 22,465 | 2336 |
| Total hip replacement constructs using an Exeter V40 stem implanted in exemplar centre censoring revisions for instability and infection | 4,121 | 2,621 | 2,030 | 655 | 69 |
| Total hip replacement constructs using constructs used at exemplar centre implanted in all other units censoring revisions for instability and infection | 86,842 | 54,445 | 32,307 | 10,171 | 689 |
| Total hip replacement constructs using constructs used at exemplar centre implanted in exemplar centre censoring revisions for instability and infection | 4,121 | 2,621 | 2,030 | 655 | 69 |
