# Supplementary material for: Factors associated with implant survival following total hip replacement surgery: A registry study of data from the National Joint Registry of England, Wales, Northern Ireland and the Isle of Man
Source: PLoS Med. 2020 Aug 31;17(8):e1003291. doi: 10.1371/journal.pmed.1003291 (PMC7458308; doi:10.1371/journal.pmed.1003291)
Supplement: Attachment — Submitted filename: V1.2_PLOS_response_to_reviewers.docx [file pmed.1003291.s010.docx]

8^th^ May 2020

Dear Ms Veitch,

**PMEDICINE-D-20-00146R1 - What makes a Total Hip Replacement last longer, the implant or who puts it in?**

**Findings from the National Joint Registry for England, Wales, Northern Ireland and the Isle of Man.**

We thank you for your helpful reviews and comments on our manuscript. We have addressed your comments and queries as detailed below and have altered the manuscript accordingly. We would be very grateful for your further review for suitability for publication in PLOS Medicine.

Thank you very much again for your time,

Yours sincerely,

Jonathan T Evans on behalf of the authors

Academic Clinical Lecturer in Trauma & Orthopaedics

University of Bristol, UK

**Requests from the editors:**

*We'd suggest using the general style for PLOS Medicine titles and therefore would simplify the second subpart of the title and just have "xxxyy: registry study" (rather than the current style which includes all the study settings).*

Thank you. We have made the change as suggested.

“What makes a Total Hip Replacement last longer, the implant or who puts it in? A registry study”

*At this stage, we ask that you include a short, non-technical Author Summary of your research to make findings accessible to a wide audience that includes both scientists and non-scientists. The Author Summary should immediately follow the Abstract in your revised manuscript. This text is subject to editorial change and should be distinct from the scientific abstract. Please see our author guidelines for more information: https://journals.plos.org/plosmedicine/s/revising-your-manuscript#loc-author-summary*

This has now been completed.

### Why Was This Study Done?

- In general, Total Hip Replacement (THR) is safe and effective at reducing pain and restoring mobility to people with end-stage arthritis of the hip.
- Different brands of hip replacement have been shown to have varying survival rates at different follow-up timepoints.
- In England and Wales in 2017 over 822 different types of hip replacement were used.
- Reducing variation in outcomes following surgery is an important aim of the National Health Service (NHS) in England and Wales.
- A national database of all hip replacements in England and Wales (the National Joint Registry) has shown variation in survival rates between different hospitals.
- A few hospitals are highlighted by the database for having better survival rates than the others.

### What Did the Researchers Do and Find?

- One of the hospitals with better survival rates for hip replacements than the others uses only one type of hip replacement for all patients.
- When this hospital was compared to everyone else using the same hip replacement, after taking the patients’ age, sex and general health into account, they no longer had better results than anyone else.

### What Do These Findings Mean?

- This suggests that the better results seen in this one hospital are not down to the skill of the surgeon, or the set-up of the hospital but in fact down to the choice of hip replacement.
- This may be the case with other brands of hip replacement that as well.
- Whether implant selection is more important than other variables, such as the surgeon, in other specialities needs further research.

*In the abstract, the findings presented currently don't present effect sizes and confidence intervals so it's hard for the reader to get a sense of what the main findings really show (for the crude and adjusted analyses), particularly as the p-value for the adjusted analysis is not stat sig by conventional cutoffs. If possible, the effect estimates and CI's from the main text should also be added here together with the p-values that are currently given.*

Thank you for highlighting this. The effect sizes for both crude and adjusted analyses have now been included in the abstract.

“Crude analyses including all THRs demonstrated better implant survival at the exemplar unit with an all-cause construct failure of 1.7% (95% CI: 1.3 to 2.3) compared to 2.9% (95% CI: 2.8 to 3.0) in the rest of the country after 13.9 years(log rank test P<0.001). The same was seen in analyses adjusted for age, sex and American Society of Anaesthesiology (ASA) score (difference in Restricted Mean Survival Time 0.12 years (95% CI 0.07 to 0.16) (P<0.001).”

*In the last sentence of the Abstract Methods and Findings section, please describe the main limitation(s) of the study's methodology.*

Thank you, we have now included this:

“The data in this study is observational and conclusions regarding causality must be interpreted with caution. Our outcome is revision surgery and whilst important, we recognise it is not the only marker of the success of a THR.”

*If possible, please change the in-text referencing style (which should be simple if referencing software was used) to giving the callouts in square brackets (eg, [1, 2] rather than superscript numbers). Many thanks*

Thank you for pointing this out, the in-text citations have now all been changed.

*Is it possible to clarify if the study had a prospective protocol or analysis plan? Please state this (either way) in the Methods section.*

*a) If a prospective analysis plan (from your funding proposal, IRB or other ethics committee submission, study protocol, or other planning document written before analyzing the data) was used in designing the study, please include the relevant prospectively written document with your revised manuscript as a Supporting Information file to be published alongside your study, and cite it in the Methods section. A legend for this file should be included at the end of your manuscript.*

*b) If no such document exists, please make sure that the Methods section transparently describes when analyses were planned, and when/why any data-driven changes to analyses took place.*

*c) In either case, changes in the analysis-- including those made in response to peer review comments-- should be identified as such in the Methods section of the paper, with rationale.*

Thank you for highlighting this to us. We have added the extra section below into the end of the methods section.

### “Planning of analyses

The analysis plan was made prior to the start of all analyses and agreed amongst co-authors. No data-driven changes to the analysis plan were made.”

**Reviewer #1:**

*Thanks for the opportunity to review your manuscript. My role is a statistical reviewer so I have concentrated on the data and analysis aspects of the manuscript, and the reporting of these. This manuscript aims to find whether individual or unit level factors lead to better outcomes in THR. I have provided overall comments, and then followed these with more specific queries. The page number refers to the page of the original word document not the compiled PDF.*

*The main aim of this study is to disentangle individual (i.e. patient characteristics and implant types) from unit level factors. The unit effect (exemplar vs. other) is estimated as a fixed effect, without accounting for the multi-level structure of the data. For this type of research question a common approach would be to estimate a multi-level model for the outcome (a shared frailty model in this case of time-to-event data) and use the random intercepts to describe the variation, and then add individual and unit level factors to estimate whether accounting for composition (patient characteristic at sites) explains differences in the outcome.*

Thank you for this observation, we agree that a multi-level model approach is one way to conduct this type of study. In this case, however, as we were interested in the effect of the implant rather than the unit itself, we elected to use the analyses presented. Whilst a multi-level model may be useful in partitioning within and between level effects, we have previously encountered difficulty getting them to converge in data sets of this size. In order to achieve convergence, we have had to simplify the shape of the baseline hazard which would potentially violate the assumptions of a Multi-level Weibull or exponential models. This model misspecification, from simplification, ultimately leads to inappropriate standard errors and has the potential for introducing bias especially in the case of non-proportional hazards. We therefore elected for a single level (the patient) model as this was more concordant with our original objectives.

*One of they key strengths of the manuscript is the large dataset from the joint replacement registry. My issues with the analysis come from the approach used to estimate unit vs individual effects and the missing data.*

*One of the fundamental parts of the study design is the selection of the RD&E unit as the reference unit for good survival outcomes for THRs. The last available report of the joint replacement registry highlighted several other units with better than expected outcomes for THR. It's not clear to me from this manuscript why just this individual unit was selected when there are apparently other high-performance units. Where these other units with good outcomes pooled with the rest of the units in the comparison of RD&E vs. other?*

Thank you for this comment. You are correct that there are other units with “better than expected” survival results and as you suggest, these were pooled with the rest of the units in the comparisons. This particular unit was selected as it is unique in that it only uses one femoral stem for all patients and as a result is not as susceptible to selection bias as units that use a range of implants with varying patterns of use seen in patients with different demographics. This was briefly mentioned (albeit not explicitly) in the methods and discussion but we agree that we need to be more explicit about the reasoning behind the decision. In view of this we have included the following paragraph in the methods section.

“The choice of the RD&E as the exposure group (rather than any of the other units with “better than expected” survival results) is due to a lack of selection bias, in that every patient in this unit receives the same femoral stem, regardless of age, sex or indication. This lack of selection bias is unique to this unit.”

*The two major problems I can see with this approach is that it doesn't account for clustering within units (so the SE will be incorrect) and that there is no way to asses the variation across all of the units and come down to comparison of one selected unit with the rest in the registry.*

We agree that there may be some imprecision in the standard error due to lack of independence between patients treated within the same unit. However, we believe intra-unit correlation is relatively small after you account for patient level effects and the choice of implants used by the surgeon. In our experience, unit-level performance is strongly mediated by the choice of implants, hence the need for the analysis performed in this paper. However, please see our comments previously around multi-level / fraility models and difficulties in large samples and convergence and the compromises required with many of the approaches. We now reflect on this in the paper.

Assessment of the variation between units is performed bi-annually and reported annually in the NJR 2018 annual report (the analyses for which are performed at our facility). These analyses allow for variation between units to be identified. Those that are above the 99.8% Control limit, which is the pre-determined threshold, are reported as having “better than expected” survival results. It is on this basis that the exemplar was chosen.

*The reporting of missing data isn't clear - ~30% of patients have missing BMI and presumably aren't included in the main analysis, but from Figure 1 I don't understand if these patients are excluded in this pathway, or if the exclusions used to create the complete case dataset are made after this point. It would be helpful for each of the covariates used in the analysis if a table reporting how many patients had missing data for each of the variables by the strata used to presented Table 1.*

Thank you for highlighting this. We can see how we have not made it completely clear when these exclusions were applied. The total dataset at the end of figure 1 did not exclude any cases based on missing co-variates. As you suggest, the exclusions made to create the complete case dataset were made after this point (at the time the potential confounder was introduced into the model and the sample size restricted as part of a sensitivity analysis). In view of this, we have included a table in the supplementary material detailing the distribution of missing data for each of the co-variates used in either the main or sensitivity analyses. Reference is now made in the main text to direct the reader to this table.

“*Missing data*

Cases missing data on potential confounders (age, sex and American Society of Anaesthesiology score (ASA), BMI or socioeconomic status) were retained in analyses that were not using that specific model as a co-variate. A table detailing the distribution of missing data on these co-variates can be seen in the supplementary material.”

Table 1 – Distribution of missing data

|  | Exemplar centre n (%) | All other units n (%) | All other units restricted to same implants n (%) |
| --- | --- | --- | --- |
| Total | 6,227 | 658,531 | 148,295 |
| Missing age | 0 (0) | 0 (0) | 0 (0) |
| Missing sex | 0 (0) | 0 (0) | 0 (0) |
| Missing ASA | 0 (0) | 0 (0) | 0 (0) |
| Missing SES | 16 (0.3) | 39,223 (6.0) | 8,569 (5.8) |
| Missing BMI | 2,277 (36.5) | 247,742 (37.6) | 45,453 (30.7) |

*A complete case analysis was used and justified as 'MI can introduce bias if not truly missing at random'. Unless done very poorly MAR approaches are also robust to when data is MCAR, and should be considered a more principled approach over complete-case analysis. The registry data does appear to have gaps in some of the variables that may make MI difficult to use (but given the description that exists it doesn't look impossible). I don't think this analysis necessarily needs to include MI, rather the limitations of a complete-case analysis should be adequately described in the discussion.*

We agree with your comments. As a result, we have included the following in the discussion regarding the limitations of complete case analysis.

“The use of complete case analysis over multiple imputation for handling missing co-variates was also a potential weakness and may result in a loss of power by restricting the sample size. Given the distribution of missing data (see supplementary information) and the large numbers offered by the registry we felt that a complete case analysis was suitable for this study and any reduction in power would be negligible.”

*P4, L31. It's probably a result of the compiled PDF but Figure 1 looks like it has some image issues (it's a bit blurry).*

Thank you for highlighting this and we apologise. We have checked the file individually and it appears clear when opened on its own.

*P5, L1. Are there multiple patient records in this dataset (i.e. sequential unilateral HR)?*

Yes, there are sequential hip replacements performed on different sides within the same patient included in this study. We treated each hip as an individual case. There is a risk of failure of one THR leading to subsequent failure of the other side in cases of infection, however given this is also the case from other joint replacements (e.g. knee) or from other conditions leading to a higher propensity to infection, we felt this risk was negligible and therefore did not exclude these from analyses.

*P5, L22. What proportion of censored outcomes were administrative (end of data availability) and how many were for death? Death is a competing risk for implant failure and it seems likely that the censoring from death and failure are not independent. Is the proportion of censoring from death small enough it is unlikely change implant failure hazard rates accounting for competing risks?*

In the study population, 79,537 cases were censored for death and 574,629 were censored administratively at the point death tracing was conducted. With regards to accounting for competing risks, this is something we have addressed previously in a different paper.^1^ Total hip replacement is not associated with an increased risk of death and therefore net survival models are appropriate for use in THR analyses.^2-5^ In brief, crude and net failure are estimating different things, one is not biased in respect to the other. In this case we are interested in net failure i.e. how long these hip replacements last in immortal patients, rather than crude failure i.e. what is the probability of the hip replacement needing revision before they die.

1.	Sayers A, Evans JT, Whitehouse MR, Blom AW. Are competing risks models appropriate to describe implant failure? Acta Orthopaedica 2018; 89(3): 256-8.

2.	Cnudde P, Rolfson O, Timperley AJ, et al. Do Patients Live Longer After THA and Is the Relative Survival Diagnosis-specific? Clinical Orthopaedics and Related Research 2018 Jun; 476(6): 1166–1175.

3.	Hunt LP, Ben-Shlomo Y, Clark EM, et al. 90-day mortality after 409 096 total hip replacements for osteoarthritis, from the National Joint Registry for England and Wales: a retrospective analysis. The Lancet 2013; 382(9898): 1097-104.

4.	Hunt LP, Ben-Shlomo Y, Whitehouse MR, Porter ML, Blom AW. The Main Cause of Death Following Primary Total Hip and Knee Replacement for Osteoarthritis: A Cohort Study of 26,766 Deaths Following 332,734 Hip Replacements and 29,802 Deaths Following 384,291 Knee Replacements. The Journal of Bone and Joint Surgery American volume 2017; 99(7): 565-75.

5.	Lie SA, Engesaeter LB, Havelin LI, Gjessing HK, Vollset SE. Mortality after total hip replacement: 0-10-year follow-up of 39,543 patients in the Norwegian Arthroplasty Register. Acta Orthop Scand 2000; 71(1): 19-27.

*P7, L26. BMI is included into the standard categories, but in my experience with patient data from joint replacement registries the number of patients with BMI >35 is much higher than seen in the general population. Is s the case for THRs in the UK? Does including the extended categories (particularly a 'super-obese' >40 category) provide additional adjustment that gives different results?*

BMI was categorised into six groups as per the World Health Organisation categories:

Table 1 - Distribution of BMI accross categories

|  | **BMI (kg/m^2^)** | **Overall** | **RD&E** | **All other units** |
| --- | --- | --- | --- | --- |
| Underweight | <18.5 | 840 (0.5) | 34 (0.6) | 806 (0.5) |
| Normal Weight | 18.5 – 24.9 | 23,506 (15.2) | 874 (14.0) | 22,632 (15.3) |
| Overweight | 25 – 29.9 | 42,755 (27.7) | 1,581 (25.4) | 41,174 (27.8) |
| Class I Obesity | 30 – 34.9 | 27,013 (17.5) | 985 (15.8) | 26,028 (17.6) |
| Class II Obesity | 35 -39.9 | 9,480 (6.1) | 350 (5.6) | 9,130 (6.2) |
| Class III Obesity | >40 | 3,198 (2.1) | 126 (2.0) | 3,072 (2.1) |
| Missing BMI data |  | 47,730 (30.9) | 2,277 (36.6) | 45,453 (30.7) |
| **Total** |  | **154,522** | **6,227** | **148,295** |

As a result, our analyses did include a BMI >40 category. The distribution of our data can be seen in the above table. Given the model including BMI was only included as a sensitivity analysis, we have not included the above table in the manuscript but can do so at the editor’s discretion.

*Supplementary Fig1 + 2. For the Supplementary material, it would be helpful if there was an explanatory section for the graphical checks of non-proportionality and GOF - a brief explanation of what the diagnostic*

Unfortunately, the end of your comment was missing. We assume you were interested in what diagnostic checks we made for non-proportionality and goodness of fit.

We have included a brief discussion of these graphs in the supplementary information as you suggest.

“The above figure demonstrates that when using an FPSA model that allows the hazard of failure to vary with time, there is a difference between the hazard of failure at the exemplar centre and in all other units (the solid lines). The fact that these solid lines cross is highly indicative of the fact that the hazards are not proportional through the entire follow up of the study.”

“Goodness of fit was assessed visually using the above figure as well as by assessment of log-likelihood of different models. A model with five knots was chosen as further knots provided more complexity with little improvement in log-likelihood.”

**Reviewer #2:**

*Thank you for the opportunity to review this work.*

*The work highlights the importance of informed and consistent surgeon choices rather than surgical skill in order to improve patient outcomes using NJR data. It demonstrates that the results achieved by a positive outlier centre are achievable by all surgeons rather than the elite few and uses robust data and methodology to do this.*

*The benefits of reducing variation and revision rates by adopting best practice are not just patient related but have far reaching cost implications on the health care system.*

*Using one centre that uses a single femoral stem and is a positive outlier in the NJR reduces bias and allows for this in-depth study and is only achievable with analysis of registry data.*

*The use of flexible parametric survival analysis by the authors is commended, however, the majority of readers may not be familiar with this method. I would therefore suggest the authors expand on this method and why it is most suitable for this study. The use of sensitivity analysis and PPI also add to the validity if the findings.*

*In particular:*

*Methods:*

*Please expand on the suitability and use of FPSA.*

Thank you for this comment, we have included a short explanation of the benefits of FPSA in the methods section. Whilst further detail may be useful for those with little experience of flexible parametrics, we feel that this may overcomplicate the relatively simple message of the article.

“FPSA modelling offers an advantage over more traditional semi-parametric techniques such as Cox regression as, in addition to allowing effects to vary with time via cubic splines, they allow us to estimate a baseline hazard function. This baseline hazard allows the estimation of absolute effects, such as survival, for both groups given certain values of covariates, rather than simply an estimate of the relative effect between the two groups (hazard ratio) as is given in Cox regression.”

*Results:*

*In the adjusted analyses section, the restricted analysis is important as it is the construct that is the most important factor rather than the individual components and there are still acceptable numbers in this restricted analysis. Consider simplifying the sentence regarding the null hypothesis with regard to RMST as it is clunky and difficult to interpret, Please expand on the the difference in RMST between groups changing over time in Figure 4, as this may not be clear to some readers.*

Thank you for highlighting this. We have made an attempt to rephrase the sentence so that it becomes clearer.

“After 13.9 years there is a discrepancy in restricted mean survival time (RMST) of 0.02 years (95% CI: -0.02, 0.07, P=0.33). A P-value of 0.33 suggests there is little or no evidence of any difference in survival of THRs after 13.9 years between those implanted in the RD&E compared to elsewhere when the same implants were used.”

We have also added a sentence explaining figure 4 in greater detail, as you suggest.

“Figure 4 shows how the difference in RMST between the two sub-groups changes over time. RMST is reported in years and estimates the difference in life expectancy of the THR between the two exposure groups i.e. the extra time a THR lasts because it was implanted in the RD&E. The difference in RMST changes slightly over time, most notably at two time points, 3 years and at 10 years. This may reflect particular modes of failure such as loosening of cups at 10 years, potentially due to cementation technique. It should be noted that for the majority of time reported the confidence intervals cross the null value of 0. Graphically it appears that for a short period there may be a transient centre-effect, however analysis of the entire period shows no such centre effect.”

*For the sensitivity analysis, the performance of the five most commonly implanted stems indicates that not all stems of the same mode of fixation are comparable. This is an important finding and may be lost in the supplementary material. Consider including in the main text or expanding on this finding.*

We agree that this is an important finding, however it is important to not overstate conclusions from this. The analyses were very crude and were simply a demonstration that other brands may achieve similar results. This is important to us as this article is a demonstration of a concept (of the importance of implant choice in general) rather than a report of the success of one mode of fixation or one particular company’s implants. More detailed comparison of constructs can be seen in a previous article from out unit using NJR data (reference below).

Deere K, Whitehouse M, Porter ML, Blom AW, Sayers A. Assessing the non-inferiority of prosthesis constructs used in hip replacement using data from the National Joint Registry of England, Wales, Northern Ireland and the Isle of Man: A Benchmarking study. BMJ Open 2019.

*Discussion:*

*The authors suggest that implant choice is responsible for the 'better than expected' results at the RD&E rather than surgeon skill. Does consistency also contribute to this and can this be measured? Are there any other centres that are as consistent in their implant choice (especially if it is a different implant) and are their results comparable? If surgeons consistently use the same implant, even if it is a poorer performing implant, do they have improved performance compared with less consistent surgeons?*

Yes, this is a very interesting and important discussion. We however felt it was beyond the scope of this study. It is a study we hope to complete soon however registry data must be analysed and reported carefully and (when reporting at a unit level and thus potentially identifiable) with the permission of the individual surgeons involved. We believe that a wider study of the effect of consistency on survival is possible and look to complete this soon. The concept of consistency with other implants is also useful and we have recently been granted permission to investigate this with another unit using a single (different) implant, although this practice has more recently been adopted and has not been place over the life of the registry as is the case here.

*In summary, this is an important study with a valuable message that has been examined using strong methodology with registry data. It identifies factors that are easily modifiable (i.e. implant choice) for a surgeon to improve implant survivorship and confirms the findings of the GIRFT initiative. It is concisely and clearly written, with appropriate methodology and conclusions, and provides novel information that is important within the discipline.*

Thank you very much for your kind words, they are much appreciated.

**Reviewer #3:**

*The authors address an important issue within the arthroplasty surgery community, what is the main driver for outcome (defined as revision) the implant or the surgeons (and his team) who puts it in.*

*The aim was to investigate whether "better than expected" survival results of one type of implant within one clinic was due to the unit or the implant.*

*For analysis they used Flexible parametric survival analysis (FPSA). The latter has the advantage of taken time-depended covariates into account, but this analysis technique may also show effects which may not be present. The authors assessed FPSA models for goodness of fit against KM curves.*

*The NJR data were complete 95% primary and 90% revision, what were those data for the RD&E unit?*

These figures for completeness of data are quoted from the first NJR data quality audit (reporting year 2014/15) and reported in the 2017 NJR annual report. They cover the entirety of the NJR and do not report on a unit level, so that information is not in the public domain. The 2019 NJR annual report offers slightly more detail (accuracy for hips and knees separately) but still does not report by unit.

*What was the minimum number of TH procedures each surgeon did at the RD&E unit. For that matter in the discussion section it could be discussed that all surgeons performed at least x number, which also excludes a surgical factor being a confounder in the outcome "revision".*

The NJR records surgeon details in two ways, who is the Consultant in Charge of the case and who was the named lead surgeon for a case. This accounts for cases performed by trainees/residents and fellows.

Of the 6,230 cases performed at the RD&E there were 18 recorded Consultants in Charge with a range of numbers of THRs from 1 to 1,919. 95% of THRs in the RD&E were under the same five Consultants in Charge.

Regarding “Lead surgeon”, there were 83 different recorded surgeons who performed a range from 1 THR to 992 THRs at the RD&E that were included in the study dataset. 68.1% of THRs were performed by one of six surgeons. We can include these data in the paper if the editors feel it will add to the manuscript and then refer to them in the discussion as suggested by the reviewer.

We believe that FPM can be considered as a generalisation of many survival models. Specifically, they are a generalisation of the exponential family of models which included Poisson and Weibull models. We also note that when the effect of interest is proportional between treatment arms the results between FPM and Cox models are indistinguishable. However, departures from proportionality are more easily detected using the FPM approach, therefore we believe this approach is less likely to find things which are not truly there.

*Strengths Weakness section*

*Please explore more e.g. why FPSA seems to better compared to KM with proportional hazard models ; competing risk analysis. But FPSA may have some disadvantage as well, it may "dictate" the survival curve as such. The latter will give more insight to the clinical reader on advantages and disadvantages of these two widely sued techniques.*

Thank you for this comment. As explained in one of the earlier reviewer comments, we feel that a competing risks model is inappropriate in these analyses as the outcome we are interested in is net failure (i.e. expected failure in an immortal cohort) as we are interested in the effect of the implant themselves. The main (commonly used) alternative to FPSA in this situation would be a Cox proportional hazards model. As we demonstrate in Figure 2 of the supplementary material when the hazard of failure is allowed to vary with time, the lines for THRs performed in each group crosses, demonstrating non-proportionality. Whilst there are ways to work around this with Cox proportional hazards modelling (namely reporting Hazard Ratios for different parts of the curves separately), the use of FPSA negates the need for this and allows us to report the results of a single model whilst also estimating a baseline hazard function which (in our opinion) makes the results easier to interpret.

We would respectfully disagree that FPSA dictates the shape of the survival curve. The model was built with a restricted cubic spline with five knots and allows the baseline model to vary its shape with time. The inclusion of time-varying coefficients for the RD&E also allows the shape of the hazard in the RD&E to be different to the rest of the country. This is one of the major advantages of FPSA over more traditional models of THR survival such as the Cox, Weibull or Poisson model whilst allowing measures of absolute effects.

The information available on the website of one of the developers of the FPM approach is useful in demonstrating this. It allows you to place and move knots in order to explore how models fit and the consequences of piecewise approaches and different types of splines. This can be included as a reference in the full manuscript if the editor wishes.

<https://pclambert.net/interactivegraphs/spline_eg/spline_eg>

*I would also recommend a statistician for review*

*Nevertheless, in conclusion I would congratulate the authors with their work*

Thank you very much for your kind words.
