# Supplementary material for: Factors associated with implant survival following total hip replacement surgery: A registry study of data from the National Joint Registry of England, Wales, Northern Ireland and the Isle of Man
Source: PLoS Med. 2020 Aug 31;17(8):e1003291. doi: 10.1371/journal.pmed.1003291 (PMC7458308; doi:10.1371/journal.pmed.1003291)
Supplement: Attachment — Submitted filename: V1.0_third_revision_responses.docx [file pmed.1003291.s011.docx]

**1. Please provide all main figures in .tif or .eps format. For more information about how to convert your figure files, please see our guidelines: http://journals.plos.org/plosbiology/s/figures. Once you've converted your files to .tif or .eps, please also make sure that your figures meet our format requirements: http://journals.plos.org/plosbiology/s/figures#loc-file-requirements / https://journals.plos.org/plosmedicine/s/figures#loc-how-to-submit-figures-and-captions**

I apologise for my misunderstanding of the file requirements. I have now changed the formats and run them all through the PACE tool. The only thing that wasn’t clear was the format of the tables. I have provided these as .docx files. I hope this is correct.

**2. Please ensure that all main figure files are clearly labeled in the File Inventory.**

This labelling has now been updated and is hopefully correct.

**3. Please indicate the corresponding author on the title page with an asterisk (*) and include the corresponding author's email address. Only an asterisk and the email address itself are required.**

I have now removed all the links to the institutions so that all that remains is the asterisk and the corresponding authors email address (plus the note about joint senior authors). I hope this is what you were referring to. I have ensured that the affiliations that did appear here are correct in the manuscript submission form.

**4. We have noted that you have the following present in your manuscript: Data Availability Statement**

**We have removed these section from your manuscript file as this information is automatically pulled from the submission form. Please make sure that the information in the submission form is correct.**

I have corrected this in line with your guidance.

**5. Please upload your Supporting Information items individually, rather than in a single file, as you refer to them individually in the text. Supporting Information files should be less than 10 MB each, and the individual figure files should be labelled clearly eg “S1 Fig”, "S1A Fig" etc.**

**Please ensure that all Supporting Information files are clearly labled in the file inventory. i.e. S1_Fig.tif, S1_Table.xlsx, S1_Text.doc, S1_Data.xlsx, S1_PRISMA_Checklist.docx, etc.**

I have now done this.

**6. Please ensure your supporting information files are individually uploaded to the File Inventory as file type 'Supporting Information' and are not present in the manuscript file.**

I have now removed the supplementary tables from the manuscript

**7. Please ensure that each supporting information file has a legend at the end of the manuscript file, after the Reference list.**

I have now checked this, and each file has a legend at the end of the manuscript.

**8. Please note that your submission cannot contain any references to generic "supporting information" files and instead must refer to specific files using our naming convention (S1 Fig, S1 Table, S1 Text, S1 Data, etc.)**

I have removed all previous references and updated them in line with your guidance.

**9. Data availability statement: Thank you for your note about the data availability statement in the response to comments. Please update the data availability statement in the manuscript submission form: “Data cannot be shared publicly. Data are available from the NJR research subcommittee researchers who meet the criteria for access to confidential data. Access to the data used in this study can be requested via njrresearch@hqip.org.uk . Full details of how to request NJR data for research can be found at: http://www.njrcentre.org.uk/njrcentre/Research/Research-requests.”**

I have included this statement in the manuscript submission form.

**10. Results and Discussion: Please refer to supporting information files by a distinct name- as Supporting Information S1 Figure, S1 Table, etc. rather than “Figure 4 of supplementary material”**

I have now made these changes.

**11. Figure 4 legend: There is a strikethrough over the word “demonstration”- please edit this sentence appropriately.**

I could not see this on my version but have retyped the legend, so hopefully this will no longer appear.

**12. S1 Table: In the table or the legend, please indicate if values are numbers and (%), if that is the case.**

I have made this change.
